# Effectiveness of adults’ spontaneous exploration while perceiving affordances for squeezing through doorways

**DOI:** 10.1371/journal.pone.0209298

**Published:** 2018-12-20

**Authors:** Eli Labinger, Jenna R. Monson, John M. Franchak

**Affiliations:** Department of Psychology, University of California Riverside, Riverside, California, United States of America; University of Minnesota, UNITED STATES

## Abstract

When motor abilities change, people need to generate information to recalibrate their perception through active *exploration*. Most prior research has focused on observers’ ability to update perception by executing experimenter-specified exploratory behaviors, however, the question of how observers spontaneously choose how to explore has been overlooked. We asked how effectively adults decide to explore when adapting to changes in their ability to squeeze through doorways. Results revealed that participants made efficient decisions about when to explore by approaching and practicing—they most often explored doorways that were near the limit of their abilities, and participants explored less often as their perceptual calibration improved. However, participants made sub-optimal decisions about how to explore, which resulted in a failure to fully recalibrate. We discuss the implications of these findings for understanding the processes of perceptual-motor recalibration that underlie real-world behavior.

## Introduction

Although the everyday task of planning and guiding motor actions may seem simple, the perceptual and motor underpinnings of selecting how to act are complex. For example, take the task of navigating through a narrow space, such as between two people standing on a crowded train car. An actor’s decision of whether to attempt to walk between the train passengers depends on the size of the space relative to the size of the actor’s body. Whether the space is wide enough relative to the actor’s body is an example of an *affordance*—the fit between an actor’s physical properties and those of the environment that allows an action to be possible [1–4]. For actors to make adaptive motor decisions, their *affordance perception* must be properly scaled to their actual motor abilities. The extant literature suggests that adults’ perception of various affordances is scaled to their abilities, including standing on and walking on slanted surfaces [5, 6], climbing up stairs and sitting on seats [2, 3, 7, 8], passing under barriers [9–13], and fitting through doorways [14–20].

Perceiving affordances is challenging because motor abilities, and thus affordances, change. When affordances change, affordance perception must be adapted to new abilities [3, 7, 18, 19]. For example, if the actor on the train puts on a backpack, perception must be rescaled to reflect the new relation between the size of the actor’s body while wearing the backpack and the space between the train passengers. This type of perceptual-motor learning is commonly referred to as *recalibration*. In laboratory studies, when participants’ leg length was altered by wearing platform shoes, participants gradually rescaled their perception of what seat heights afford sitting [3, 7, 8]. Recalibration means learning about the intrinsic relation between body and environment as opposed to learning about extrinsic environmental properties. For example, Mark [3] demonstrated that participants’ recalibration of affordance perception in the sitting task was independent of their perception of the size of the platform shoes—an extrinsic property that altered affordances. Other work showed that affordance perception and recalibration cannot be reduced to a calculation between lower-order, extrinsic properties: Judgments of affordances for reaching with a stick improved over trials despite unchanged perception of stick length [21]. Thus, affordance recalibration is a particular type of perceptual-motor learning in which observers learn to detect the intrinsic relation between their body and the environment for a particular action.

Such learning occurs through information-gathering actions known as *exploratory behaviors* [22, 23]. Exploratory behaviors are a wide range of movements that generate perceptual information, including both subtle movements, such as visually inspecting an obstacle from a distance [24, 25] or altering postural movements [8, 11, 26, 27], as well as gross movements, such as touching a surface with hands or feet [28–30], locomotion [7, 11, 18], or even practicing the target action [7, 18–20]. For some affordances, subtle forms of exploration such as postural sway—movements of the body while standing in place—are sufficient for recalibration. Postural sway generates optic flow information that specifies whether some body-scaled affordances are possible or impossible. Such postural movements are of such small magnitude that they are not detectable to the eye or a standard video camera, but must be measured using high-resolution motion trackers. A careful series of studies showed that observers generate information to recalibrate perception of affordances in the sitting task by altering postural sway movements [7, 8]. Similarly, postural sway movements of novice wheelchair users is integral in their judgments of whether they can navigate under barriers that vary in height [11, 26, 27]. In these examples, specific *practice* performing the action in question—sitting on seats while wearing the platform shoes or rolling under barriers in the wheelchair—did not confer any advantage for recalibration beyond simple movement experience. Participants who had *general movement experience* (such as spent time wheeling around the laboratory or walking in platform shoes) made judgments that were as accurate as participants who practiced. For the purposes this paper, the term practice will always refer to performing the target; in the sitting task, practice means actually sitting on seats, whereas in the barrier task practice refers to rolling under a barrier in the wheelchair.

Recent evidence suggests that exploratory behaviors required for recalibration are specific to the particular task. Whereas practice is not required for recalibrating to changes in affordances for sitting or navigating under barriers, it is required to adapt to changes in squeezing through doorways [17–19]. After putting on a backpack that altered affordances for fitting through doorways, participants made inaccurate judgments—the doorway size participants judged to be the smallest they could squeeze through differed from their actual affordance thresholds (i.e., smallest doorway they could successfully navigate) by 6-8 cm [18]. Unlike the sitting task, in which participants gradually improved over trials through visual and postural information from a distance, such forms of exploration did not result in any changes in judgments for the squeezing task [19]. However, twenty trials of practice (i.e., actually walking up to and squeezing through various doorway widths while wearing the backpack) provided feedback about which doorway widths were possible and impossible and recalibrated perception: Errors decreased to 2-3 cm. Moreover, participants who received only general movement experience (walking around the room without practicing) failed to recalibrate—errors were unchanged. Further work indicated that as few as five practice trials are sufficient to recalibrate [19], but for practice to recalibrate perception participants needed to experience both successful and failed experiences [19, 20, 31]. Thus, the existing work suggests that in the squeezing task (unlike the sitting and overhead barrier tasks) practice leads to recalibration, but other forms of exploration, such as looking, making postural adjustments, and locomoting are insufficient in the absence of practice feedback.

If different exploratory behaviors are required for different tasks, how do people deal with the real-life challenge of deciding how to explore? Most likely, observers do not make conscious decisions to engage in subtle and relatively cost-free forms of exploration such as looking from a distance or adjusting postural movements. However, when those means of exploration are insufficient for a particular task, when and how do people decide to engage in costlier forms of exploration such as locomotion and/or practice? In the current study, we address this question using the doorway squeezing task in which practice supports recalibration but visual inspection from a distance, postural movements while standing in place, and general movement experience (e.g., walking around) do not. We propose that when affordances change, a series of subtasks must be completed for recalibration to occur: 1) deciding whether/when to use gross forms of exploration versus relying on visual/postural information from a distance, 2) executing the selected exploratory behavior, and finally 3) updating affordance perception based on the consequent information. If affordance perception has not adequately recalibrated, observers may choose to continue this process until their perception is satisfactory. For example, if relying solely on visual information fails to improve calibration, observers may eventually decide expend the effort to practice the action. The latter two subtasks are well-studied, however, relatively little adult research has addressed the first subtask because most studies assign participants to conditions that specify and/or restrict how they should explore. Whereas perceptual learning studies, such as those of dynamic touch [32–34], test how spontaneous exploration calibrates perception, the same is not true of studies of affordance perception. For example, studies of recalibration to platform shoes dictate what type of exploration is permitted in different conditions [3, 7]. Similarly, studies of recalibration to squeezing through doorways while wearing a backpack manipulated what exploration was permitted in different conditions [17–19].

What information might guide actors’ decisions about whether to use gross, costly forms of exploration? It is clear from past work that adults’ confidence ratings of their affordance judgments closely track actual affordances [2, 6, 15, 28, 29, 35–37]. For example, for steps much higher than participants’ *affordance thresholds* (sometimes referred to as *critical boundaries*)—the highest step that is possible to walk up—participants easily recognize that the step is too high and are confident in their perception [2]. Likewise, participants give strong confidence ratings for steps that are much shorter than threshold and clearly steppable. However, confidence is lowest for steps nearest to threshold. Since confidence judgments show that participants recognize when they are most uncertain about affordance perception, it seems likely that participants will decide to use more costly forms of exploration, such as practicing, more often when near threshold (most ambiguous) and less often at the extremes. At the extremes, affordances that are clearly possible/impossible may be easily and more efficiently distinguished from visual information from a distance. Indeed, several developmental studies of affordance perception have measured spontaneous gross exploration and found that infants and children explore more often by approaching, touching, and shifting body positions near affordance thresholds and less often at the extremes when walking down slopes [38], descending steps [39], crossing bridges [25], stepping along ledges [40], and reaching through openings [30]. For example, infants faced with bridges of varying width almost always looked at bridges at the start of each trial [25]. However, on riskier bridges, after looking infants subsequently approached and sometimes touched the bridge, thus reserving costlier forms of exploration for when affordances were most ambiguous.

However, these developmental studies are limited because it is unknown whether the exploratory behaviors that infants and children exhibit actually improve their perceptual calibration. This is pertinent to the question of deciding *how* to explore appropriately for a given task. For example, infants who more often explored by both looking and touching when navigating across bridges of varying widths made no better decisions than infants who touched less frequently [25]. When deciding whether to reach through openings, children engaged in more exploratory touching for intermediate-sized openings compared to smaller openings [30]. For smaller openings, children were more likely to simply look at the opening and refuse to attempt. However, it is unknown whether touching behavior is more effective than simply looking—it may or may not actually improve perceptual calibration in the reaching task, but this has not been tested.

The current study addressed this shortcoming by using the doorway squeezing task, in which practice is known to facilitate recalibration but visual information from a distance and/or general locomotor exploration do not. When allowed to explore however they wish, will adults choose the appropriate means of exploration (practice) or will they rely on ineffective means of exploration (e.g., standing in place to look and/or adjust postural movements, approaching the doorway without practicing)? Although it is possible that adults might always choose to practice, this seems unlikely for two reasons. First, practicing the action is more effortful compared to other options like standing in place or taking a few steps towards the doorway. If adults believe that other means of exploration are effective (or are confident in their perception), practice might not appear to be worth the effort when less energetically-demanding options are available. Second, prior work suggests that adults might not practice because they want to avoid making motor errors. Whereas children often chose to practice and make mistakes in the doorway squeezing task, adults were more cautious which resulted in less practice experience [31]. Consequently, judgment errors were greater in participants who chose not to practice compared to those that did practice.

### Current study

The overall goal of the current study was to determine the effectiveness of adults’ spontaneous exploration for recalibrating perception in the doorway squeezing task while wearing a backpack that altered affordances. Because observers generally make accurate judgments in this task when their abilities are unaltered [16, 31], the backpack manipulation was necessary to perturb perception by altering affordances, thus creating a need for exploration in the service of recalibrating perception. Our study had two aims. First, we tested when and how often participants chose to spontaneously explore by approaching or by practicing squeezing through doorways of different sizes versus staying at the starting line and relying on visual/postural information from a distance. Second, we asked how well participants’ self-selected exploration (e.g., their decisions to explore by approaching and practicing) led to recalibration of affordance perception.

A *spontaneous exploration phase* addressed the first aim. On each trial, participants were shown a doorway and asked to make a yes/no judgment about whether they could fit through, and we measured participants’ decisions to *approach*—walking up to the doorway—and to *practice*—actually attempting to squeeze through the doorway. We compared how often participants approached and practiced for doorways that were near their affordance thresholds, that is, the smallest doorway they could successfully fit through, compared to doorways that were much smaller and much larger. As in developmental work [25, 30, 38–40], we predicted that participants would make effective decisions about when to explore by rarely approaching/practicing when presented with doorways much smaller/larger (for which visual information should be sufficient to make a judgment) but would more frequently explore intermediate ones by approaching/practicing. We also measured how participants’ confidence in their judgments varied by doorway size to determine whether participants chose to explore most often when they were the least confident. We asked whether gross exploration changed over time by comparing approach and practice rates over three successive blocks of spontaneous exploration trials. Because prior work shows that as few as five practice trials is sufficient for participants to fully recalibrate in the doorway squeezing task [19], we predicted that participants would approach and practice frequently in the first block of trials and, if initial practice led to recalibration, would seldom use such costly forms of exploration in the subsequent two trial blocks.

Additionally, we tested how restricting versus motivating exploration affected participants’ decisions to engage in gross exploration by comparing three conditions: *Practice Prohibited* (PP) participants were allowed to explore in any way except for practicing squeezing through doorways, *Practice Allowed* (PA) participants were allowed to explore in any way including practicing, and *Practice Reward* (PR) participants were allowed to practice but also received a monetary reward for making accurate judgments to provide extra motivation to explore. Assuming that participants recognize that practice is the only effective way to recalibrate in this task, we predicted that participants in the PP condition who were not allowed to practice would be less confident and would less often approach the doorway since they were not permitted a viable way to recalibrate their affordance perception. Furthermore, we predicted that participants in the PR condition would be more motivated to improve their perception and would explore more often than was necessary.

The second aim of the current study was to determine how effectively participants’ spontaneous exploration recalibrated affordance perception. We measured *affordance judgments* using a method of adjustment (MoA) procedure in which participants indicated the smallest doorway they perceived to be possible to squeeze through. *Judgment errors* were calculated by comparing affordance judgments to each participant’s actual affordance threshold. Participants provided three sets of judgments. The *Pretest* phase tested participants’ accuracy after putting on the backpack but before engaging in spontaneous exploration. The *Posttest-E* phase tested accuracy following spontaneous exploration. If participants explored effectively, we predicted that errors would decrease from Pretest to Posttest-E, but only for those participants permitted to practice (PA and PR). Finally, the *Posttest-F* phase tested participants’ accuracy after they completed 15 *Forced Practice* trials modeled after past work [17–19] that dictated participants’ exploration by assigning a fixed amount of practice. If participants were effective at practicing during spontaneous exploration, errors should not decrease from Posttest-E to Posttest-F. However, if participants failed to fully recalibrate after spontaneous exploration, we predict a decrease in error from Posttest-E to Posttest-F.

## Materials and methods

### Participants and experimental design

The final sample included 90 participants (54 female, 36 male) who were undergraduate college students aged 16.6 to 31.4 years (*M* = 19.8, *SD* = 1.9). Thirty participants were randomly assigned to each of three experimental conditions: PP (practice-prohibited; 17 female, 13 male), PA (practice-allowed; 22 female, 8 male), and PR (practice-reward; 15 female, 15 male). Participants enrolled in the study for course credit through the research participation subject pool used by the psychology department at the University of California, Riverside. Participants were required to have normal or corrected-to-normal vision and to be able to walk without assistance. Written consent was obtained from each participant (for minors, the legal guardian provided consent and the participant provided written assent). The study’s protocol (HS-15-044 “Adult decision-making for walking through doorways”) was approved by the Institutional Review Board of the University of California, Riverside.

Because participants’ decisions to spontaneously practice was a main variable of interest, it was critical that participants understood whether they were allowed to practice in each condition. Participants completed a survey at the end of the study that assessed whether they understood which behaviors they were allowed to do or were prohibited from doing during the study. Data from three participants (not included in the final sample of 90) were excluded for failure to understand what was allowed during the spontaneous exploration phase based on their survey responses. Eight additional participants were recruited but their data were excluded: Five participants failed to follow the study instructions (e.g., forced the doorway open, misunderstood the confidence rating scale), two participants were excluded because of equipment problems, and one participant chose to withdraw from the study due to illness.

### Apparatus

**Fig 1:**
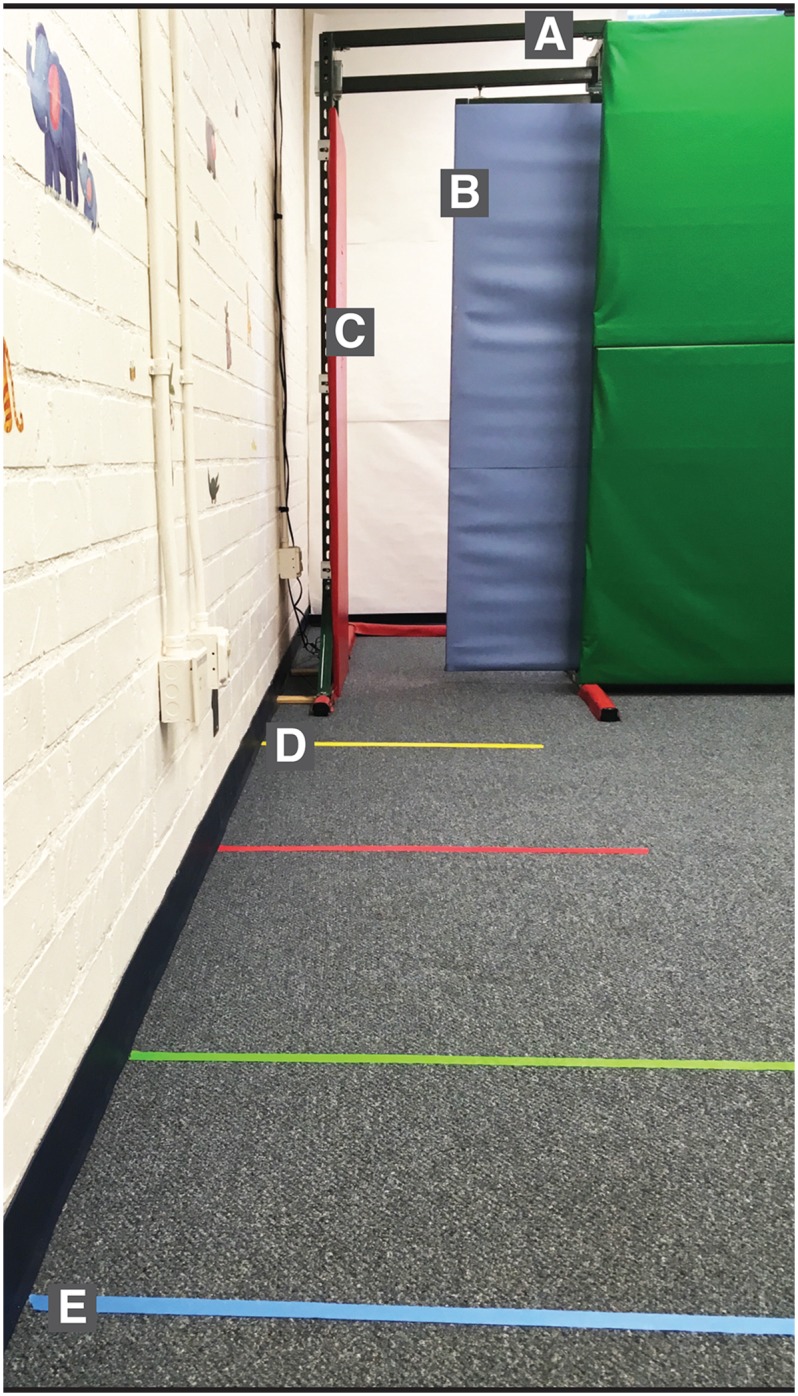
Adjustable doorway apparatus. A: Track that supported the sliding door. B: Sliding door (blue). C: Stationary panel (red). D: Yellow line used for coding approach (1 m from doorway). E: Blue starting line (3.5 m from doorway). Red and green lines were not used for the present study.

The doorway apparatus and backpack were the same as used in previous work [18, 19]. A freestanding metal framework (213 cm tall × 280 cm wide) supported a track (Fig 1A) on which a sliding door (Fig 1B) was mounted. A stationary panel (Fig 1C; 182 cm tall × 62 cm wide) on one side of the structure formed a surface perpendicular to the sliding door. The doorway’s height was 191 cm tall from the floor to the track on which the door was mounted. When opened completely, the doorway was 70 cm wide. During the course of each session, the experimenter manipulated the width of the doorway while standing behind the apparatus, out of sight of the participant. A monitor on the experimenter’s side of the doorway displayed readings from a measurement camera attached to the sliding door, allowing the experimenter to accurately adjust the width of the doorway in 0.5-cm increments. The sliding door was equipped with a locking mechanism which ensured that the doorway remained at the same width while participants attempted to squeeze through.

A fixed side-view camera recorded video and audio for subsequent coding of participants’ exploratory behaviors by providing a view of participants’ approach and entry into the doorway. To facilitate coding whether participants approached the doorway, a line 1 m from the doorway was marked in yellow tape (Fig 1D). A line 3.5 m from the doorway marked the location that participants stood at the beginning of each trial (Fig 1E).

Participants wore a backpack throughout the duration of the study. The backpack was 43 cm tall × 25 cm wide × 12 cm in depth, weighed 1.1 kg, and contained a stack of rigid cardboard to prevent compression as participants squeezed through the doorway. The backpack was secured with straps around the chest and waist to ensure that it remained centered on participants’ backs.

### Procedure

Participants wore the backpack throughout the entire study, which lasted approximately 45 minutes. Before putting on the backpack, participants were given an opportunity to visually and manually inspect it. Regardless of condition, each participant completed five experimental phases in the same order: 1) pretest judgment (*Pretest*), 2) spontaneous exploration, 3) posttest judgment following exploration (*Posttest-E*), 4) forced practice, and 5) posttest judgment following forced practice (*Posttest-F*). The three judgment phases (Pretest, Posttest-E, and Posttest-F) measured participants’ affordance perception and confidence across varying levels of experience with exploring affordances for squeezing through the doorway. Accordingly, the procedure for measuring accuracy and confidence was identical for each of the three judgment phases (see Judgment Phases section, below). Pretest judgments measured perception before any locomotor exploratory experience had been incurred. Posttest-E measured perception following spontaneous exploration (see Spontaneous Exploration section, below). Posttest-F measured perception following the forced doorway fitting practice phase (see Forced Practice section, below).

#### Judgment phases

Each judgment phase consisted of four method of adjustment (MoA) trials [3, 7, 19]. During each MoA trial, the participant stood at the starting line and the experimenter gradually moved the door in one direction until the participant said that they believed that the doorway was the smallest they could successfully squeeze through while wearing the backpack. Participants were allowed to adjust their response by telling the experimenter to increase or decrease the doorway size until they were satisfied that it was the smallest possible doorway that could be navigated. A successful passage through the doorway was defined as completely passing through the doorway in a sideways position (with the back against the stationary panel) while wearing the backpack. Participants’ bodies and the backpack were allowed to make contact with the sliding door and the stationary panel during passage. The direction of the door’s movement alternated between ascending (closed to open) on odd-numbered trials and descending (open to closed) on even-numbered trials. Because past work found no changes in affordance judgments over the course of 24 successive MoA pretest trials in the doorway squeezing task [19], four trials were deemed sufficient to accurately determine participants’ affordance perception at each phase.

Participants in the PR condition were rewarded 5 USD at the end of the study if their absolute error was less than or equal to 2.5 cm in Posttest-E; participants in the PP and PA conditions were not rewarded.

Participants made a confidence rating following the fourth judgment trial using a Likert scale rating from 1 (not at all confident) to 7 (extremely confident) as in past work [2, 6, 15]. Ratings indicated how confident participants were that their fourth MoA judgment reflected the smallest doorway width they could successfully squeeze through.

#### Spontaneous exploration phase

In the spontaneous exploration phase, participants were presented with a range of different doorway widths and were permitted to explore in any way they believed would help them decide whether it was possible to fit through each doorway. Participants began each trial at the starting line and faced away from the apparatus so that they would not see the doorway change between trials. At the start of each trial, participants were cued to turn around and face the doorway. On each trial, participants gave a yes/no response indicating whether they believed they could fit sideways through the doorway while wearing the backpack. Participants were allotted unlimited time to explore the doorway before making the judgment. There were a few necessary restrictions placed on exploration: Across conditions, participants were not permitted to manually adjust the width of the doorway or to remove the backpack. In addition, since participants in the PP condition were restricted from practicing, those participants were told that they could not attempt to fit through or move past the doorway with any part of their bodies. Otherwise, participants were permitted to explore in any way they wished. After each ‘yes’ or ‘no’ response, participants were asked to rate their confidence that their response was correct, using the same scale of 1-7 that was used in the judgment phases. The instructions read to participants at the beginning of the spontaneous exploration phase were as follows:

In the next part of the experiment, I will show you a series of thirty doorways of various sizes. Your task will be to tell me ‘Yes,’ if you think you can squeeze through the doorway, or ‘No,’ if you do not think you can squeeze through. On each trial, before answering ‘yes’ or ‘no,’ you may take time to explore or experiment with the backpack or your surroundings. You may walk around the room, touch the backpack or the doorway apparatus, lean against the wall, [*PA and PR conditions*, try squeezing through the doorway], or do anything else you think might help you make the most accurate judgment possible. [*PA and PR conditions*, The one restriction is that you may not take the backpack off]. [*PP condition*, The only restrictions are that you may not take the backpack off or attempt to move through the doorway]. You are encouraged to spend as much time as you need to make an accurate judgment, but you are not required to do anything. When you have decided whether or not you think you can squeeze through each doorway, please clearly state your response aloud. Immediately after you give your yes/no response, please tell me, on a scale of 1-7, how confident you are that the yes/no response you just gave is correct. A response of ‘1’ will indicate that you are ‘Not at all Confident’ and a response of ‘7’ will indicate that you are ‘Extremely Confident.’

Participants completed three blocks of 10 spontaneous exploration trials. In each block of trials, participants were presented with the same 10 doorway widths (16, 19, 22, 25, 28, 31, 34, 37, 40, and 43 cm) presented in a randomized order. Each participant’s affordance threshold was later confirmed to be within this range (affordance thresholds ranged from 21.1 cm to 35.6 cm), meaning that all participants were exposed to both possible and impossible doorways during the exploration phase.

#### Forced practice phase

The forced practice phase served two purposes. First, having participants practice fitting through doorways yielded a measurement of their actual affordances; measuring actual abilities was necessary for calculating judgment accuracy. Second, the forced practice phase gave all participants the same amount of practice experience regardless of how much they chose to (or were permitted to) practice during the spontaneous exploration phase. Participants completed 15 forced practice trials during which they attempted to squeeze through doorways of various widths. On each trial, participants were presented with a single doorway and were asked to attempt to fit through. On the first trial, the doorway for all participants was 25 cm wide. On subsequent trials, doorway widths were presented using a staircase method: Successful attempts to fit through the doorway were followed by a doorway measuring 2 cm narrower on the next trial; failed attempts were followed by a doorway measuring 1.5 cm wider on the next trial. The staircase procedure ensured that participants experienced both success and failure feedback by providing doorways narrower and wider than the smallest they could fit through.

### Data coding and processing

#### Practice and approach

Approach and practice behaviors were coded from visual and audio recordings using Datavyu software (datavyu.org). For every spontaneous exploration trial, coders determined whether participants *approached* to within 1 m of the doorway (one foot planted entirely across the yellow line in Fig 1D) and whether participants *practiced* by, at minimum, placing their shoulder into the doorway. A primary coder scored 100% of the spontaneous exploration trials and a secondary coder scored 25% of trials across participants to assess inter-rater reliability. Practice was not coded for participants in the PP condition who were prohibited from practicing. Coders agreed on approach for 98.9% of trials (*κ* = .98) and on practice for 98.1% of trials (*κ* = .93).

#### Affordance thresholds and judgment errors

Each participant’s *affordance threshold* was defined as the smallest doorway successfully navigated during the forced practice phase. Judgment errors in the Pretest, Posttest-E, and Posttest-F phases were calculated based on the correspondence between judgments and affordance thresholds. Each participant’s four MoA judgments were averaged to provide a single measure of affordance perception at each phase. *Absolute judgment error* was calculated by taking the absolute value of the difference between the affordance judgment and the actual affordance threshold for each judgment phase for each participant.

## Results

Two sets of analyses were conducted. The first set of analyses tested how, when, and how often participants spontaneously explored in the spontaneous exploration phase. The second set of analyses compared the effectiveness of spontaneous exploration by comparing judgment accuracy and confidence before spontaneous exploration, after spontaneous exploration, and after forced practice. For each analysis, linear mixed models (LMMs) were calculated in *R* [41] using the *lme4* package [42] with random intercepts for each participant. Significance of main effects and interactions were calculated using *F* tests with degrees of freedom determined by the Satterthwaite approximation [43, 44] using the *lmerTest* package [45]. Follow-up pairwise comparisons and trend contrasts used the Holm-Bonferroni correction to adjust for multiple comparisons. Preliminary analyses that included sex as a factor did not reveal any significant sex differences, so sex was dropped as a factor in the results reported below.

### Decisions to explore by approaching or practicing

During the spontaneous exploration phase, participants across conditions often chose to approach to within 1 m of the doorway (*M* = 58.7% of trials, *SD* = 30.0). Of the 90 participants, 86 approached the doorway on at least one trial; two participants in the PP condition and two participants in the PR condition never approached. Overall, participants who were allowed to practice (PA and PR conditions) chose to practice on *M* = 34% of trials (*SD* = 23.3). Of the 60 participants in the practice conditions, 52 practiced on at least one trial; three participants in the PA condition and five participants in the PR condition never practiced. It was surprising that these eight participants never practiced despite explicit instructions stating that they were allowed to practice. Each of the eight participants (like all participants in the sample) verified through the survey that they knew they were allowed to practice, yet they did not do so, even some participants in the PR condition for whom accuracy was rewarded. For those participants who did choose to practice, most approaches to the doorway resulted in practicing fitting through: Of the 57.8% of trials that PA and PR participants approached, slightly more than half (*M* = 57.2%, *SD* = 26.5) resulted in practice.

The following two sections examine exploration in greater detail by determining how decisions to approach and practice depended on the affordances presented by each doorway, changed over the session, and related to confidence in judging affordances.

#### How did decisions to approach and practice relate to affordances and confidence?

To test whether the affordances provided by doorways of different widths influenced participants’ confidence in their perception and their decisions to explore, doorways were grouped into five doorway width bins to capture variations in affordances. However, affordances varied greatly between participants—affordance thresholds ranged from 21.1 cm to 35.6 cm—meaning that the same absolute doorway size provided different affordances depending on the participant. Thus, *relative doorway width* was calculated to equate affordances from different doorway widths by subtracting each participant’s affordance threshold from the doorway width on each trial. A relative doorway width of 0 cm represented the smallest doorway the participant successfully navigated. *Negative* relative doorway widths represented narrow doorways that were *impossible* to fit through, whereas *positive* relative doorway widths represented doorways that were *possible* to fit through. Trials were grouped into the five bins based on relative doorway width in the following manner: *much smaller* than threshold (≤ −6 cm), *slightly smaller* than threshold (> −6 cm & ≤ −2 cm), *near threshold* (> −2 cm & < + 2 cm), *slightly larger* than threshold (≥ + 2 cm & < + 6 cm), and *much larger* than threshold (≥ + 6 cm). For each bin, we calculated the participant’s average confidence rating, approach rate (proportion of trials approaching within 1 m of the doorway), and practice rate (proportion of trials practicing fitting into the doorway, PA and PR participants only).

**Fig 2:**
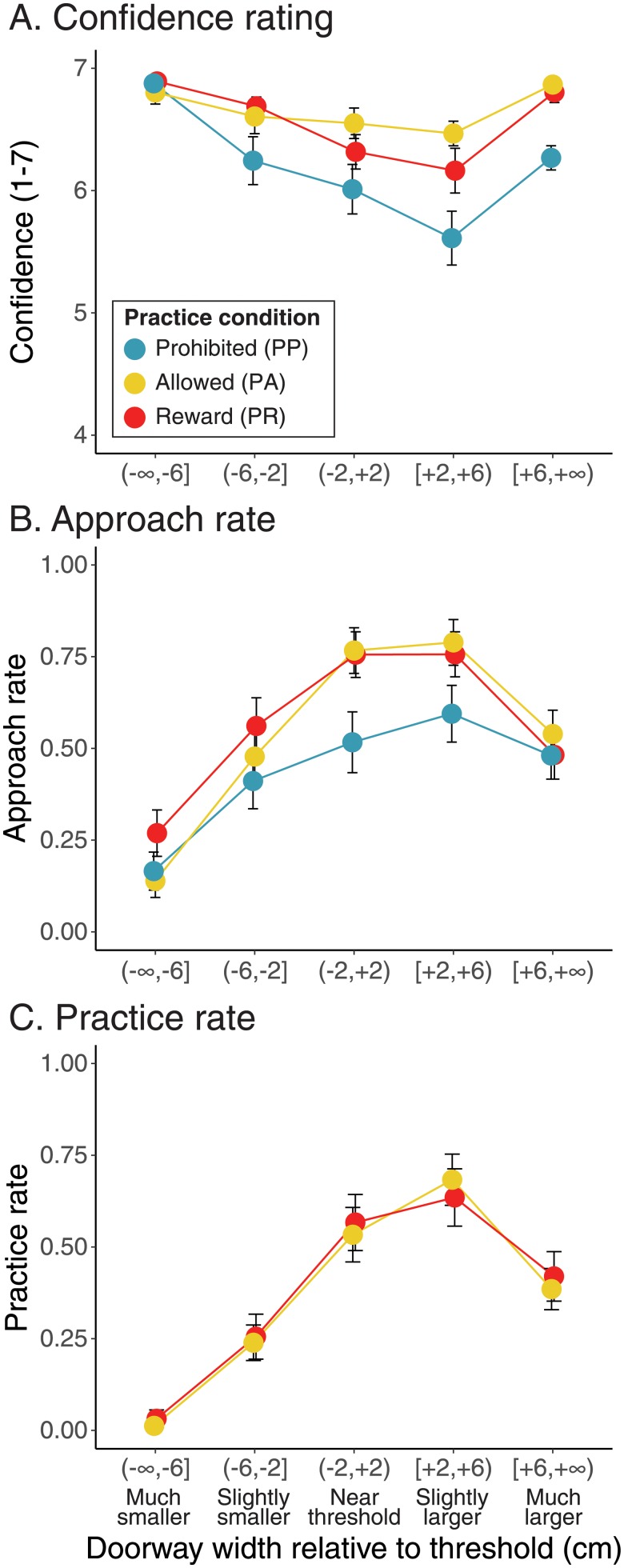
Confidence, approach, and practice by relative doorway width. X-axis shows relative doorway width bins. Positive doorway widths indicate possible doorways (larger than each participant’s affordance threshold) whereas negative doorway widths indicate impossible doorways (smaller than threshold). Symbol color indicates experimental condition (PP: teal, PA: yellow, PR: red). Error bars show ±1 SE. Data within each bin are offset horizontally for clarity of illustration. A: Mean confidence rating. B: Approach rate. C: Practice rate.

Fig 2A shows that confidence was greatest for doorways much smaller or much larger than threshold but confidence was lower for doorways near and slightly larger than threshold (see Table 1 for descriptive statistics). Furthermore, confidence was lower among participants prohibited (PP) from practicing compared with those participants allowed to practice (PA and PR), particularly for doorways near and slightly larger than threshold. A LMM predicting confidence ratings from condition (PP, PA, and PR) and doorway width bin (much smaller, slightly smaller, near threshold, slightly larger, much larger) confirmed these observations. Condition, doorway width bin, and their interaction were entered as fixed factors and participant was entered as a random factor. Table 2 shows that the effects of condition, bin, and the condition×bin interaction were all significant. To follow up on the significant effect of bin, quadratic trend contrasts by bin were calculated within each condition. Significant quadratic contrasts for each condition confirmed participants were more confident when responding to more extreme doorways (much larger or much smaller than threshold) but were less confident when responding to doorways near threshold (*p*s < .024). To follow up on the condition effect and the condition×bin interaction, pairwise comparisons were performed comparing the three conditions within each bin. Confidence did not significantly differ between the three conditions for doorways slightly smaller and much smaller than threshold. For doorways near threshold, participants in the PP condition were significantly less confident compared with those in the PA condition (*p* = .01), but no significant differences were found when comparing PP to PR or PA to PR. For doorways slightly larger and much larger than threshold, participants in the PP condition were significantly less confident compared to those in the PA and PR conditions, (*p*s < .009); no significant differences were found between PA and PR participants.

**Table 1:** Means and standard deviations for confidence ratings, approach rates, and practice rates by doorway width bin and condition.

Bin	Condition	Confidence	Approach	Practice
Much smaller	PP	6.88 (0.22)	0.17 (0.28)	
	PA	6.80 (0.49)	0.14 (0.24)	0.01 (0.06)
	PR	6.89 (0.21)	0.27 (0.33)	0.03 (0.13)
Slightly smaller	PP	6.24 (1.07)	0.41 (0.41)	
	PA	6.61 (0.77)	0.48 (0.36)	0.24 (0.27)
	PR	6.69 (0.41)	0.56 (0.42)	0.26 (0.34)
Near threshold	PP	6.01 (1.11)	0.52 (0.45)	
	PA	6.55 (0.68)	0.77 (0.34)	0.53 (0.41)
	PR	6.32 (0.77)	0.76 (0.34)	0.57 (0.42)
Slightly larger	PP	5.61 (1.21)	0.59 (0.42)	
	PA	6.47 (0.55)	0.79 (0.34)	0.68 (0.38)
	PR	6.16 (1.00)	0.76 (0.34)	0.63 (0.43)
Much larger	PP	6.27 (0.54)	0.48 (0.35)	
	PA	6.87 (0.23)	0.54 (0.35)	0.39 (0.31)
	PR	6.80 (0.46)	0.48 (0.36)	0.42 (0.37)

**Table 2:** Summary of LMM results predicting confidence ratings, approach rates, and practice rates from condition and doorway width bin. Degrees of freedom and resulting *p* values obtained using the Satterthwaite approximation. Note: practice analysis excludes the Practice Prohibited condition.

	*SS*	*MSE*	*df*	*F*	*p*	
**Confidence**						
Condition	2.53	1.27	2, 87.61	6.87	.0017	**
Bin	15.03	3.76	4, 326.31	20.39	<.0001	***
Condition × Bin	3.71	0.46	8, 326.19	2.51	.0115	*
**Approach**						
Condition	0.16	0.08	2, 86.93	1.61	.2064	n.s.
Bin	15.12	3.78	4, 342.24	73.88	<.0001	***
Condition × Bin	0.98	0.12	8, 342.24	2.38	.0164	*
**Practice**						
Condition	0.00	0.00	1, 58.15	0.01	.9066	n.s.
Bin	15.08	3.77	4, 230.32	61.99	<.0001	***
Condition × Bin	0.08	0.02	4, 230.32	0.33	.8559	n.s.

*p<.05,

**p<.01,

***p<.001

Participants’ decisions to explore by approaching the doorway mirrored confidence ratings—participants most often approached doorways they judged with less confidence. Fig 2B shows that participants rarely approached doorways much smaller or much larger than threshold (for which they could readily decide using visual information from a distance) but frequently approached doorways near threshold (see Table 1 for descriptive statistics). Approach rates were similar between the conditions for doorways at the extremes, but for doorways near threshold PA and PR participants more often approached compared to those in the PP condition who were prohibited from practicing. As with confidence, a LMM predicting approach rate from condition (PP, PA, and PR) and doorway width bin (much smaller, slightly smaller, near threshold, slightly larger, much larger) revealed a significant effect of bin and a significant condition×bin interaction (Table 2). Unlike confidence, there was no significant main effect of condition. Quadratic trend contrasts for each condition confirmed that participants more often approached intermediate doorways compared to doorways at either extreme (*p*s < .0001). To follow up on the condition×bin interaction, pairwise comparisons were performed comparing approach rates between conditions within each bin. For doorways near threshold, participants in the PP condition approached less often compared with participants in both the PA and PR conditions (*p*s < .0246); approach rates did not differ between the PA and PR conditions. No significant differences between conditions were found at the other bins.

Practice rates for participants in the PA and PR conditions closely matched approach rates and were nearly identical between the two conditions (Fig 2C, Table 1). A LMM predicting practice rates from condition and doorway width bin revealed only a significant effect of bin (Table 2). Quadratic trend contrasts confirmed that participants practiced less often for extreme doorways and more often for intermediate doorways in both conditions (*p*s < .0001).

#### How did confidence, approach, and practice change over time?

To determine how participants’ confidence, approach, and practice changed over time during the spontaneous exploration phase, average confidence ratings, approach rates, and practice rates were calculated across the 10 trials in each of the three trial blocks. Because each participant received the same 10 doorway widths in each block, changes in aggregated behaviors across blocks are meaningful. It should be noted that each block of 10 trials provided different affordances for participants depending on their individual threshold. For example, a participant with a threshold of 26 cm would have received 4 impossible doorways and 6 possible doorways in each block, whereas a participant with a threshold of 32 would have received 6 impossible doorways and 4 possible doorways. However, affordance thresholds were distributed similarly across the three conditions (PP: *M* = 28.0 cm, *SD* = 3.0; PA: *M* = 26.8 cm, *SD* = 3.6; PR: *M* = 28.0 cm, *SD* = 3.9), suggesting that an uneven distribution of possible and impossible doorways should not bias comparisons between conditions.

**Fig 3:**
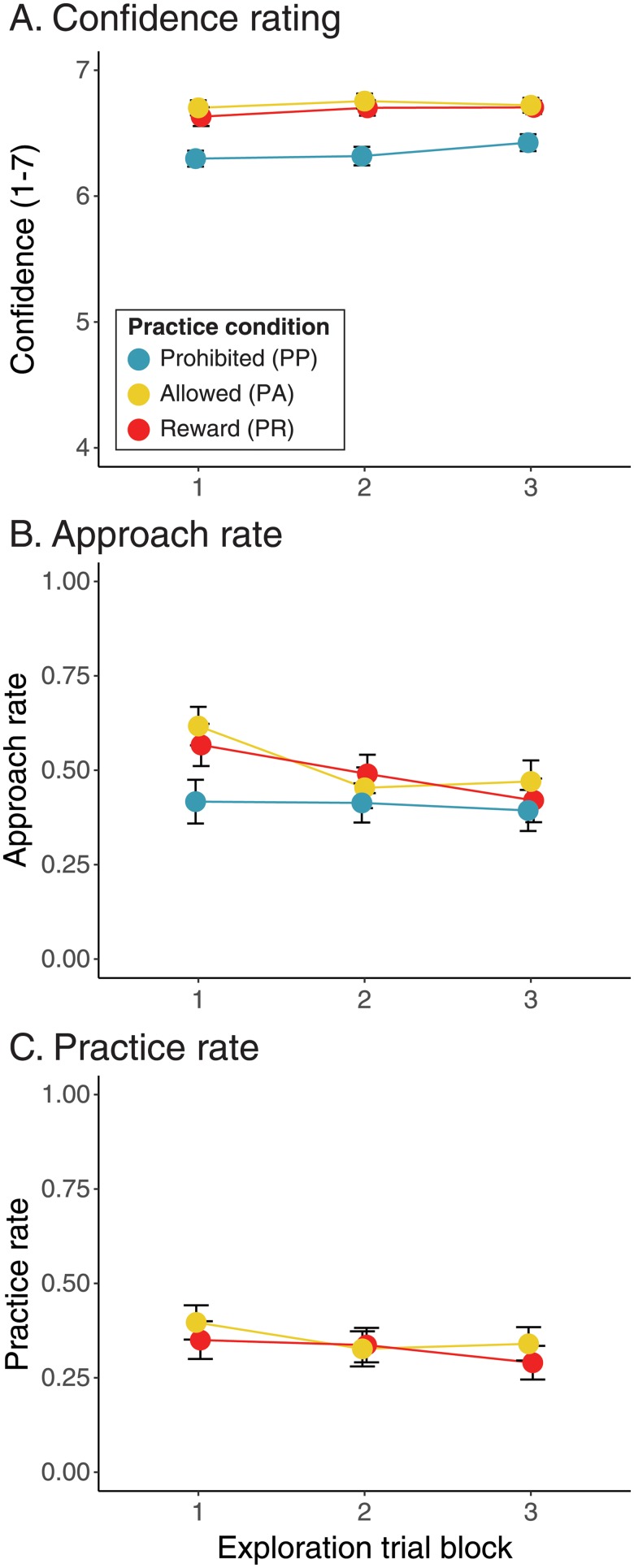
Confidence, approach, and practice by trial block. Symbol color indicates experimental condition (PP: teal, PA: yellow, PR: red). Error bars show ±1 SE. Data within each trial block are offset horizontally for clarity of illustration. A: Mean confidence rating. B: Approach rate. C: Practice rate.

Fig 3A shows that confidence increased slightly over blocks regardless of condition, however, confidence was greater across blocks for those participants who were allowed to practice (see Table 3 for descriptive statistics). This was confirmed in a LMM on confidence ratings with condition (PP, PA, PR), trial block (1, 2, 3), and the condition×trial block interaction as fixed effects and participant as a random effect. The results summarized in Table 4 show that there were significant effects of condition and trial block on confidence but no significant interaction. A linear trend contrast testing the effect of trial block (collapsing across conditions) indicated a significant increase in confidence (*p* = .0033) over time. Pairwise comparisons between conditions (collapsing across trial blocks) indicated that PP participants were less confident overall compared to those in the PA (*p* = .0001) and PR (*p* = .0003) conditions but that confidence did not differ between the PA and PR conditions (*p* = .5795).

**Table 3:** Means and standard deviations for confidence ratings, approach rates, and practice rates by trial block and condition.

Block	Condition	Confidence	Approach	Practice
1	PP	6.30 (0.35)	0.42 (0.32)	
	PA	6.70 (0.32)	0.62 (0.28)	0.40 (0.25)
	PR	6.63 (0.41)	0.57 (0.31)	0.35 (0.27)
2	PP	6.32 (0.40)	0.41 (0.28)	
	PA	6.75 (0.33)	0.45 (0.30)	0.33 (0.25)
	PR	6.70 (0.34)	0.49 (0.28)	0.34 (0.25)
3	PP	6.42 (0.37)	0.39 (0.30)	
	PA	6.72 (0.32)	0.47 (0.31)	0.34 (0.24)
	PR	6.70 (0.28)	0.42 (0.32)	0.29 (0.24)

**Table 4:** Summary of LMM results predicting confidence ratings, doorway approach, and practice from condition and trial block. Degrees of freedom and resulting *p* values obtained using the Satterthwaite approximation. Note: practice analysis excludes the Practice Prohibited condition.

	*SS*	*MSE*	*df*	*F*	*p*	
**Confidence**						
Condition	0.57	0.29	2, 87	12.13	<.0001	***
Block	0.25	0.12	22, 174	5.26	.0060	**
Condition × Block	0.18	0.04	4, 174	1.85	.1215	n.s.
**Approach**						
Condition	0.03	0.02	22, 87	1.18	.3133	n.s.
Block	0.55	0.27	2, 174	19.52	<.0001	***
Condition × Block	0.27	0.07	4, 174	4.75	.0012	**
**Practice**						
Condition	0.00	0.00	1, 58	0.23	.6352	n.s.
Block	0.11	0.05	2, 116	4.20	.0174	*
Condition × Block	0.03	0.02	2, 116	1.32	.2708	n.s.

*p<.05,

**p<.01,

***p<.001

Fig 3B and Table 3 show that participants in the PA and PR conditions approached more often in the first block but approach rates declined in blocks 2 and 3. In contrast, PP participants approached at the same rate throughout the exploration phase. A LMM predicting approach rates from condition and trial block revealed a significant main effect of block and a significant condition×block interaction (Table 4). Follow up linear contrasts were conducted within each condition to determine whether approach changed over the session. There was no significant change in approach rates for the PP condition (*p* = .8946), but approach rates decreased significantly in the PA and PR conditions (*p*s < .0001).

Fig 3C shows that practice rates decreased slightly over the course of the exploration phase for participants in the PA and PR conditions (see Table 3 for descriptives). A LMM predicting practice rates from condition (PA, PR) and trial block revealed only a significant main effect of trial block (Table 4). A follow-up linear contrast on trial block (collapsed across conditions) suggested a significant decrease in practice over trials (*p* = .0116).

### Effectiveness of spontaneous exploration in recalibrating perception

The first set of analyses showed that although participants made efficient decisions about when to approach and practice, they often failed to choose the most effective means of exploration (practicing). The second set of analyses tested the extent to which participants’ decisions to approach and to practice were effective for recalibrating perception by comparing judgment errors and confidence before the spontaneous exploration phase (Pretest), after the spontaneous exploration phase (Posttest-E), and after all participants were forced to practice squeezing through doorways (Posttest-F).

#### Judgment error

**Fig 4:**
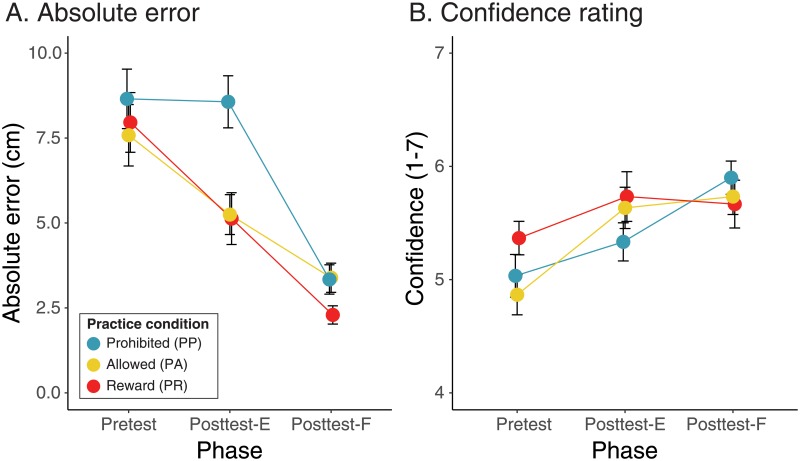
Absolute judgment error and confidence ratings by phase. Symbol color indicates experimental condition (PP: teal, PA: yellow, PR: red). Error bars show ±1 SE. Data within each trial block are offset horizontally for clarity of illustration. A: Mean absolute judgment error. B: Mean confidence rating.

Fig 4A shows changes in absolute error by phase and condition (see Table 5 for descriptive statistics). At pretest, errors were large and roughly equivalent across the three conditions. Change in errors from Pretest to Posttest-E depended on practice: Errors decreased for participants in the practice conditions (PA and PR) but were unchanged for participants in the PP condition who were not allowed to practice. Across conditions, errors decreased from Posttest-E to Posttest-F following forced practice, suggesting that participants did not fully recalibrate from spontaneous exploration. These findings were confirmed in a LMM that predicted absolute errors from condition (PP, PA, PR), phase (Pretest, Posttest-E, Posttest-F), and the condition×phase interaction as fixed effects with participant as a random effect. Table 6 shows that the LMM revealed significant condition, phase, and condition×phase effects.

**Table 5:** Mean and standard deviations for absolute error and confidence by phase and condition.

Phase	Condition	Error	Confidence
Pretest	PP	8.65 (4.79)	5.03 (1.03)
	PA	7.58 (4.96)	4.87 (0.97)
	PR	7.96 (4.81)	5.37 (0.81)
Posttest-E	PP	8.57 (4.20)	5.33 (0.92)
	PA	5.25 (3.22)	5.63 (1.00)
	PR	5.13 (4.18)	5.73 (1.20)
Posttest-F	PP	3.34 (2.37)	5.90 (0.80)
	PA	3.39 (2.35)	5.73 (0.87)
	PR	2.29 (1.48)	5.67 (1.15)

**Table 6:** Summary of LMM results predicting absolute errors and confidence ratings from condition and phase. Degrees of freedom and resulting *p* values obtained using the Satterthwaite approximation.

	*SS*	*MSE*	*df*	*F*	*p*	
**Absolute error**						
Condition	78.19	39.09	2, 87	3.60	.0314	*
Phase	1188.03	594.02	2, 174	54.74	<.0001	***
Condition × Phase	114.73	28.68	4, 174	2.64	.0353	*
**Confidence**						
Condition	0.59	0.29	2, 87	0.51	.6021	n.s.
Phase	21.83	10.91	2, 174	19.00	<.0001	***
Condition × Phase	5.57	1.39	4, 174	2.42	.0499	*

*p<.05,

**p<.01,

***p<.001

Two sets of follow-up pairwise comparisons were conducted to better understand the condition×phase interaction. The first set tested for differences between conditions within each phase: At Pretest and at Posttest-F there were no significant differences between the three conditions. However, at Posttest-E participants prohibited from practicing (PP) made significantly larger errors compared with those in the practice conditions (PA and PR) (*p*s < .0017); there was no difference between the two practice conditions at Posttest-E. The second set of pairwise comparisons tested for significant decreases in error between successive phases within each condition: Participants in the practice conditions showed significant decreases in error from Pretest to Posttest-E (*p*s < .0134) and from Posttest-E to Posttest-F (*p*s < .0001). In contrast, errors in the PP condition did not significantly decrease from Pretest to Posttest-E (*p* = .9190) but did decrease from Posttest-E to Posttest-F (*p*< .0001).

#### Confidence ratings

Fig 4B shows that confidence ratings increased slightly over the three phases (see Table 5 for descriptive statistics). A LMM on confidence ratings with fixed effects of condition (PP, PA, PR) and phase (Pretest, Posttest-E, Posttest-F) was calculated with participant as a random effect (Table 6). The LMM confirmed the effect of phase but also revealed a condition×phase effect that just reached significance. To follow up on the main effect of phase, pairwise comparisons (collapsed across conditions) showed that confidence improved from Pretest to Posttest-E (*p* = .0001) but the change from Posttest-E to Posttest-F did not reach significance (*p* = .0784). To follow up on the interaction, pairwise comparisons between conditions within each phase were calculated, however, no comparison revealed a significant effect (*p*s > .1506), which was unsurprising given the marginally significant interaction.

## Discussion

The current study investigated the effectiveness of adults’ spontaneous exploration in an affordance recalibration task—squeezing through doorways after putting on a backpack. Although some aspects of the exploratory process were effective, others were not. Adults efficiently chose to explore by approaching and practicing most often for doorways near the limits of their abilities (and those for which they were least confident). For doorways that were much larger or smaller than threshold, participants were more likely to simply respond after looking at the doorway from the starting line. Furthermore, participants approached and practiced more often earlier in the study, but did so less often after judgment accuracy (and confidence) improved over the course of the session. Other findings revealed ineffective exploration: Participants’ decisions about how to explore were sub-optimal—participants who were allowed to practice often approached without practicing or simply stood at the starting line to visually inspect the doorway from a distance, two types of exploration that are inappropriate for recalibration in the squeezing task. Lacking sufficient practice experience, participants failed to fully recalibrate after the spontaneous exploration phase. Full recalibration occurred only after a block of trials in which participants were required to practice on every trial. This discrepancy—poor recalibration following spontaneous exploration versus effective recalibration after forced practice—suggests that adults’ everyday (spontaneous) perceptual-motor recalibration might not be as optimal as what is observed in laboratory tasks that dictate how participants should explore.

### What accounts for ineffective exploration?

The most surprising finding in the current study was that adults who were allowed to (and sometimes did) practice failed to fully recalibrate. Participants practiced less frequently than expected: They approached and practiced on only 34% of spontaneous exploration trials. On many trials, adults explored in ways that do not support recalibration in this task: On 24% of trials they approached without practicing and on 42% of trials they did not even approach (perhaps relying on visual/postural information from a distance). One explanation that can be ruled out is that participants did not know they were allowed to practice. Almost every participant in the PA and PR conditions did practice at least once, and those that never practiced indicated in the post-study questionnaire that they were allowed to practice.

There are several possible explanations for why adults practiced infrequently. First, participants may have been reluctant to expend the effort to practice, especially if they believed their perception was accurate. Indeed, across conditions, confidence ratings were uniformly high, even in the pretest judgment phase when all groups made large judgment errors. Overconfidence may have led participants to rely on less costly forms of exploration, such as visually inspecting the doorway from the starting line. Moreover, participants’ confidence failed to track changes in calibration: Confidence increased for PP participants following spontaneous exploration even though their judgment accuracy did not change. Second, participants may have been reluctant to engage in exploration that would result in error. Consistent with other work [31], adults were more likely to practice fitting through larger, possible doorways as compared with smaller, impossible doorways. Because participants must experience both successful and failed practice outcomes [19, 20, 31], skewing practice towards possible rather than impossible doorways may have left some participants with insufficient failure experience. Third, participants may not have been sufficiently motivated to perform well in the task, resulting in little impetus to explore. Of the three explanations, this is the least plausible because participants who had the potential of earning a reward for accuracy still did not practice more often than those who were not rewarded (consequently, only 11/30 PR participants actually received the reward by reducing errors to below 2.5 cm). However, we cannot rule out the possibility that the potential reward (5 USD) was not sufficiently motivating.

A potential flaw in an explanation based on low *quantity* of practice is that past work found that as few as five practice trials (albeit ones that provided both success and failure experience) were sufficient to recalibrate perception in this task [19]. For participants in the PA and PR conditions, practicing on 34% of 30 trials meant that, on average, they practiced about 10 times. At face value, this should be enough practice (unless practice was skewed towards success experiences, as mentioned above). An alternative explanation is that the *quality* of practice during the spontaneous exploration trials differed in comparison to forced practice. Anecdotally, we observed that participants sometimes practiced by gently inserting a shoulder into a doorway, but then retracted the shoulder without pushing the body into the doorway as far as it could go. However, when asked to fit through the doorway in the forced practice block participants appeared to make a more concerted effort to squeeze through, pushing the body into the doorway with greater force. Because we could not devise a reliable way to operationalize how much effort participants put into practicing, both “low effort” and “high effort” attempts were coded equivalently. Thus, if participants’ spontaneous practice attempts were halfhearted, they might have failed to generate useful feedback about success and failure.

Regardless of which of the above explanations account for adults’ ineffective exploration, the fact remains that what adults *chose to do* during the spontaneous exploration phase was insufficient to fully recalibrate perception. This finding suggests that prior studies showing adults’ consistent recalibration from practice in this task [17–19] may not generalize as well to real-world situations as previously thought. Rather, we predict that adults would be likely to err when faced with this task in everyday life. Future work should study spontaneous recalibration in other affordance tasks to determine how likely adults’ self-determined exploration and recalibration matches the limits observed in more controlled laboratory situations.

### Implications of studying the real-time process of exploration

More broadly, the current study speaks to the need to study the entire process of recalibration. We proposed that the process of recalibration involves three subtasks: 1) deciding how to explore, 2) executing exploratory behaviors, 3) updating perception. Previous research with adults has exclusively tested how participants execute exploratory behaviors to update affordance perception under conditions that dictate what types of recalibration are and are not allowed. The current study is novel in allowing adults to choose how to explore. Given the preceding section, it is clear that adults’ ability to execute exploratory behaviors and update perception alone does not guarantee recalibration if they fail to make appropriate decisions about how to explore.

Like infants and children in past work [25, 30, 38–40], adults in the current study made appropriate decisions about when to use gross means of exploration. Across conditions, participants most often practiced for doorways that were near threshold or slightly larger, but rarely approached/practiced when doorways were much smaller or much larger than threshold. Adults’ confidence ratings mirrored exploration, suggesting that adults’ assessment of their own perceptual calibration was related to their exploratory decisions. Note, however, that although adults practiced at appropriate times, they also explored with inappropriate means (approaching without practicing) at appropriate times, suggesting a dissociation between knowing when more exploration is needed versus knowing what type of exploration is needed. Of course, that adults chose to practice at all suggests that they did believe (correctly) that practice would aid recalibration. Furthermore, participants in the practice prohibited condition less often approached the doorway compared with the other two conditions, suggesting that on some level they were aware that approaching the doorway would not be fruitful if they could not practice.

Past developmental work shows that infants use a variety of exploratory behaviors—shifting positions, touching with hands, rubbing with feet—when faced with different affordance tasks [25, 30, 38–40]. However, with few exceptions [25], prior work has not addressed which of these behaviors are actually effective at improving perceptual calibration in the studied tasks. The current study goes a step beyond past developmental work by showing that although adults (like infants and children) explore at the right time, they do not always use appropriate means of exploration. Finding that adults choose the right time to explore but often do so ineffectively raises the possibility that infants and children in past work are also ineffectively exploring. A consistent finding in infant affordance studies is that infants’ decisions do not improve over the course of the session [46, 47]. One possible explanation for this finding is that although infants are exploring, they are not choosing the appropriate exploratory behaviors so they fail to improve despite repeated exploration. However, if those exploratory behaviors proved to be appropriate, the problem might reside in infants’ ability to update perception. Indeed, recent work shows that young children (aged 4-7) practice appropriately in the doorway squeezing task but fail to update perception [31], suggesting a deficit in the ability to learn from practice information.

The current study was novel in showing that adults change how they explore over time as their affordance perception becomes better calibrated, concurrent with increases in confidence. Adults in the PA and PR conditions practiced and approached less often following the first block of trials. Participants in the PP condition approached at the same rate in each of the three blocks of trials, suggesting that they continued to do so since their perception did not improve (although, counterintuitively, their confidence did increase). An intriguing direction in future work would be to test adults’ spontaneous exploration and recalibration in the stepping, sitting, and overhead barrier tasks [3, 7, 8, 11]—tasks in which recalibration occurs gradually over the course of trials. Would participants recognize that their recalibration is slow and continue to explore the same way until achieving a particular level of calibration? Or, would their confidence rapidly increase (out of line with perception), leading to an undesirable change in exploration?

Comparing the stepping/sitting/barrier tasks to the squeezing task used in the current study might also shed light on the extent to which the exploratory subtasks we proposed are under implicit or explicit control (and whether that varies across different affordance tasks). Our framing of the first subtask as a decision—deciding how to explore—implies an explicit process, which seems appropriate with reference to practicing an action, something participants are surely aware of doing. However, it is less clear whether participants in the stepping/sitting/barrier tasks make conscious decisions to alter their postural sway in specific ways. Whereas confidence in perception provides a potential source of information for participants to make explicit decisions about exploration, it is unclear what information might underlie the implicit control of other types of exploration, such as postural sway. Lacking systematic testing across different tasks, we remain agnostic about whether these processes are implicit or explicit.

### Exploration for different affordances

The current study adds to a growing body of literature showing that the appropriate means of exploration differ across various affordance tasks. Notably, recent research suggests that practice is required for recalibration in the doorway squeezing task [17–19], but other studies show that practice is not required for the sitting and barrier tasks [7, 11]. In past work using the squeezing task, it is difficult to make a clear-cut case that practice is required because it is impossible to exhaustively test (and rule out) every conceivable type of exploratory behavior. For example, participants did not recalibrate after walking around the lab and pressing the backpack against a wall [18], but might they have recalibrated if they were instead instructed to spin in circles, heft the backpack, and/or turn the body to the side while making judgments? While the current study also cannot eliminate such possibilities (because they were not explicitly tested), it does demonstrate that adults failed to *spontaneously discover* any means of exploration aside from practice that led to recalibration. Participants in the practice prohibited condition approached the doorway often and tried many different things. Anecdotally, they sometimes walked up to the door and turned their bodies sideways as if to compare their body size to the doorway size, and many participants touched the doorway or the backpack with their hands. However, regardless of how participants in the practice prohibited condition explored, they did not recalibrate.

Although such findings cannot confirm that practice is required in the squeezing task, they suggest that practice is the only effective means of exploration that adults spontaneously select and execute. Furthermore, the results (replicating previous findings) confirm that several exploratory means available to participants in the PP condition were *insufficient* for learning. First, visual information from a distance while standing at the starting line—such as optic flow generated from postural movements—was available to all participants while making judgments in the pretest and both posttest phases as well as while exploring. However, such visual information was not sufficient for recalibration when practice was prohibited. Second, whereas in past work general locomotor was sufficient to recalibrate in the wheelchair task [11], participants in the PP condition did not recalibrate despite frequently generating locomotor experience while approaching the doorway.

We note that since participants always wore backpacks on their backs and only viewed the backpack at the start of the study before putting it on, lack of visual information about the backpack is a potential candidate that could contribute to participants’ errors across conditions. We think this is unlikely for several reasons. First, prior work using the same task systematically compared accuracy between participants wearing the backpack on the front of the body, providing continual visual information about the backpack, to participants wearing the backpack on the backs [18]. Although errors were significantly smaller for participants who could see the backpack, the effect size was small and both groups of participants made large errors before receiving practice experience regardless of visual experience. Second, prior studies of stepping while wearing platform shoes and reaching while holding a tool have demonstrated that affordance judgments are independent of perceptual judgments about the size of the platform shoes or tool [3, 21]. Thus, it is unlikely that participants’ perception of the backpack itself accounted for the errors observed in the current study.

## Conclusion

The current study demonstrates the need to study the entire process of exploration—how actors decide when and how to explore—not just their ability to recalibrate. We found that even though actors made good decisions about when use particular forms of exploration, their choices about how to explore were ineffective and precluded recalibration of perception. Finding a dissociation between actors’ decisions about when to explore and how to explore suggests that the different subtasks in the process of exploration may be independent. Developmental evidence supports this as well: Even though infants are adept at choosing when to use gross exploratory behaviors, children aged 4 to 12 show deficits in updating perception based on exploratory practice [31]. More work is needed to determine the effectiveness of the entire exploration-recalibration process for other affordances. In doing so, we might better understand how laboratory studies of affordance perception generalize to actors’ real-life performance in everyday motor tasks.
